# Effect of Solvents on the Electrical and Morphological Characteristics of Polymer Solar Cells

**DOI:** 10.3390/polym11020228

**Published:** 2019-02-01

**Authors:** Jun Young Kim

**Affiliations:** Department of Semiconductor Engineering, Engineering Research Institute (ERI), Gyeongsang National University, Jinju 52828, Korea; kimjy86@gnu.ac.kr; Tel.: +82-55-772-1732

**Keywords:** Inverted polymer solar cell, bulk heterojunction, solvent effect, crystallization, morphology, impedance

## Abstract

The nanoscale morphology of poly(3-hexylthiophene) (P3HT) and [6,6]-phenyl-C_71._ butyric acid methylester (PCBM) blend film is affected by various parameters such as the solvent, coating, and thermal annealing conditions. We investigated the effect of solvents on the performance of inverted solar cells based on the active layer of a P3HT:PCBM bulk heterojunction. P3HT and PCBM (weight ratio 1:0.8) were dissolved in chlorobenzene (CB) and dichlorobenzene (DCB). The difference in the volatility characteristics of the solvents resulted in different P3HT crystallite morphologies. The difference in the P3HT:PCBM film morphology was systemically investigated via atomic force microscopy, ultraviolet (UV)-visible absorption spectroscopy, X-ray diffraction, and electrical impedance spectroscopy. The DCB solvent lead to better P3HT crystallinity and device performance. For example, the short-circuit current density (J_SC_) and the power conversion efficiency (PCE) of the device using DCB (9.89 mA/cm^2^ and 3.62%, respectively) were larger than those (9.12 mA/cm^2^ and 3.01%) of the device using CB.

## 1. Introduction

Polymer solar cells (PSCs) using the bulk heterojunction (BHJ) structure of a conjugated polymer and a fullerene derivative have many advantages, such as low fabrication cost, light weight, and flexibility [1,2]. Over the last decade, their power conversion efficiency (PCE) has increased significantly [3]. Recent remarkable progress in PSCs has been realized via the synthesis of new low bandgap polymers, non-fullerene acceptors, and tandem structures of polymer/acceptor BHJ architecture, which have yielded an efficiency of ~14% [4,5,6]. This sharp improvement in PSCs suggests that a PSC efficiency of ~20% for commercialization can be achieved in the near future [7]. However, the stability of PSCs has become increasingly important, because the commercial solar cell panels require both high PCE and long-term stability [8].

Among the efforts to increase the power conversion efficiency (PCE), many studies have focused on improving the nanoscale BHJ morphology of the photo-active layer [9,10,11,12,13,14,15,16,17,18,19]. Recently, the morphology has been improved by aligning the energy level through the inter-polymer ternary system, and the use of an alloy acceptor system [20,21]. In addition, the thermal and solvent annealing process can yield simple control of the polymer/acceptor BHJ system morphology. Thermal annealing has been extensively used to increase the ordering of polymer conjugation chains, thereby increasing the charge carrier mobility and the light absorption [9,10,11,12,13]. Solvent annealing can also improve the polymer crystallinity and device performance [22,23].

In this work, we have investigated effective and simple morphology control of polymer/acceptor BHJ systems through solvent annealing. Especially, the analysis of the influence of morphology control on electrical characteristics examined the cause of efficiency increase and decrease. This is the novelty of this study, which will be used as a measure to analyze the influence of numerous morphological changes of organic solar cells on electrical properties in the future. Polymer solar cells based on the BHJ blend of poly(3-hexylthiophene) (P3HT) and [6,6]-phenyl-C_71_ butyric acid methyl ester (PCBM) with a 1:0.8 weight ratio were considered. We compared chlorobenzene (CB) and dichlorobenzene (DCB) as their differing solvent volatility can lead to different P3HT crystallinity. The nanoscale morphology of the P3HT:PCBM film was investigated via atomic force microscopy (AFM), UV-visible absorption spectroscopy, X-ray diffraction (XRD), and electrical impedance spectroscopy.

## 2. Experimental Methods

**Figure 1:**
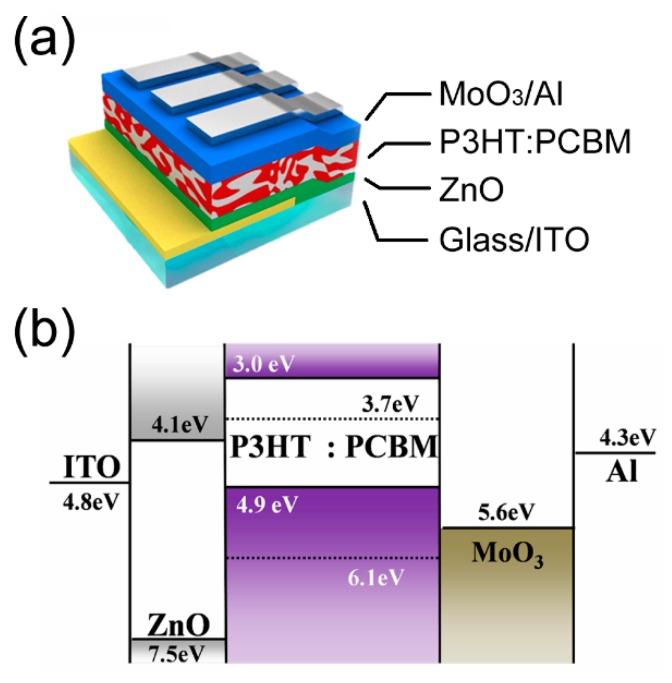
(**a**) Schematic of device structure and (**b**) energy level diagram of inverted P3HT:PCBM solar cells.

Devices were prepared on glass/patterned ITO (Indium Tin Oxide) substrates (thickness: ~150 nm, sheet resistance: ~10 Ω/square. The substrates were cleaned using isopropyl alcohol, de-ionized water, and acetone, in an ultrasonic bath, and dried in an oven. For efficient electron extraction, zinc oxide nanoparticles (ZnO NPs) dispersed in butanol were spin-coated on top of the ITO substrate. The ZnO NPs were prepared in accordance with a previously reported method [24]. Then, a P3HT:PCBM (1:0.8 weight ratio, thickness: 120 nm) film was spin-coated from the solution of P3HT (Rieke Metals, 4002-E, *M*_w_ ~60,000 g/mol, regioregularity ~94%, polydispersity ~1.5) and PCBM (ADS). We optimized the device characteristics by changing the concentration of the P3HT:PCBM blend ratio and the annealing conditions for CB and DCB. For the CB solution, we used a concentration of 3 wt % and thermal annealing at 150 °C for 30 min. For DCB, a concentration of 1.5 wt % and thermal annealing at 150 °C for 15 min were employed. All processes for preparing the solution and thermal annealing of the film proceeded in a glove box environment filled with argon gas. The MoO_3_ (10 nm)/Al (100 nm) were thermally evaporated in the vacuum chamber under a pressure of ~10^−6^ Torr. Figure 1 shows the device structure and energy level diagram of the inverted PSCs. The device area overlapped by ITO and Al was 0.2 cm^2^. Ultraviolet (UV)-visible absorption spectra and X-ray diffraction (XRD) patterns were obtained from the P3HT:PCBM films, which were fabricated on the cleaned quartz substrates.

Current density–voltage (J–V) characteristics under illumination (100 mW/cm^2^) from a 300 W solar simulator (Newport 91160A, Newport Corporation, USA) were measured in vacuum by using a Keithley 237 source measure unit. The incident photon to charge carrier efficiency (IPCE) spectra were measured by using a lock-in amplifier (Model 7265, Signal Recovery, AMETEK Scientfic Instruments, UK) when the devices were illuminated by a monochromatic light from a xenon lamp through the monochromator (SpectraPro-150, Acton Research Corporation, USA). The UV-visible absorption spectra, XRD patterns, and surface morphology were measured by using a DU-70 spectrophotometer (Beckman, USA), M18XHF-SR (MAC Science Co., Japan), and a non-contact atomic force microscope (AFM, Park Systems XE-100, Korea). Moreover, impedance spectroscopy (frequency range: 100 Hz–10 MHz) was performed using a HP-4192A impedance analyzer (Agilent Technologies, USA).

## 3. Results and Discussion

**Figure 2:**
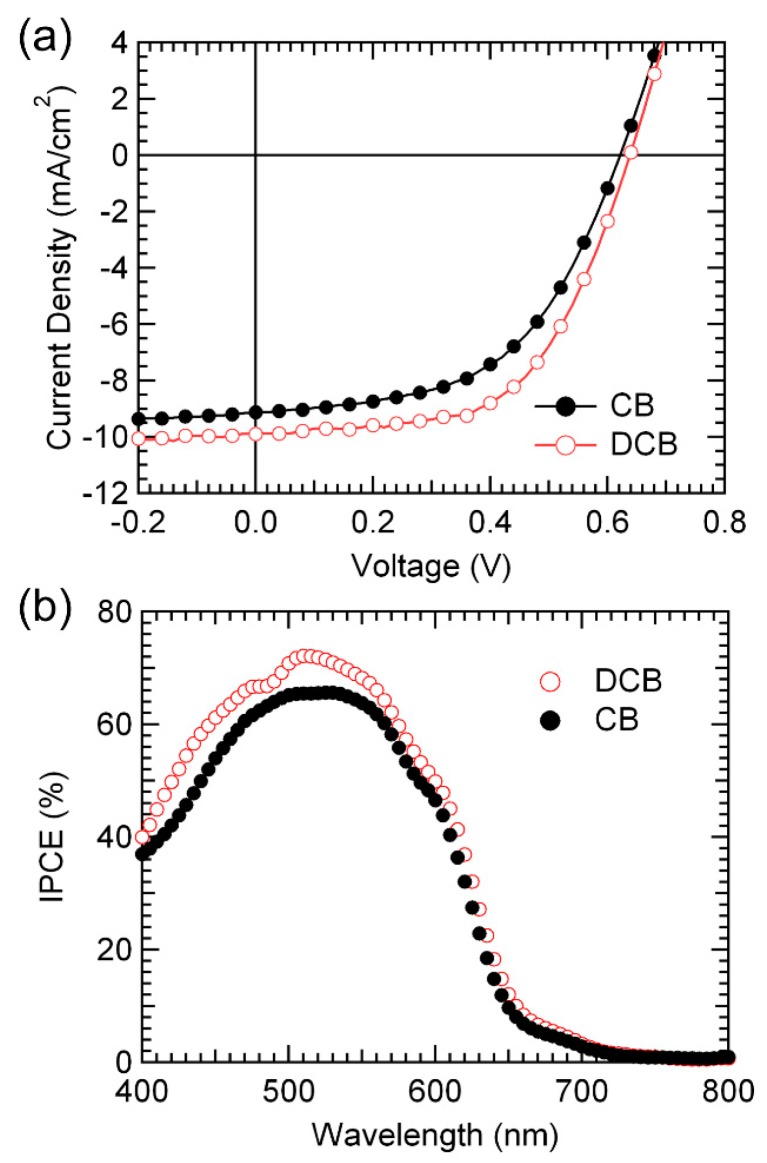
(**a**) J–V characteristics under 100 mW/cm^2^ illumination and (**b**) IPCE spectra of inverted P3HT:PCBM solar cells using CB and DCB solvents.

The effect of the CB and DCB solvents was revealed by comparing the J–V and IPCE characteristics of the two PSC devices (see Figure 2). Under 100 mW/cm^2^ illumination, an open-circuit voltage (V_OC_), and a short-circuit current (J_SC_), fill factor (FF), and power conversion efficiency (PCE) of 0.62 V, 9.12 mA/cm^2^, 0.53, and 3.01%, respectively, are obtained for the device using CB. Corresponding values of 0.64 V, 9.89 mA/cm^2^, 0.57, and 3.62% are obtained for the device using DCB. All of these performance parameters are for best samples, and each device is made of five samples with an average PCE value of 2.97% using CB-based device, 3.57% using DCB-based device. Although the P3HT:PCBM using CB (~150 nm) is thicker than that using DCB (~120 nm), the characteristics of the device using DCB are superior to those of the device using CB. This results from the fact that, in the P3HT: PCBM system using the DCB solvent, the optical effect of light absorption to the active layer is maximized [25,26]. In other words, the amount of light absorbed increases with the thickness of the polymer, but the optical effect that yields the optimal short-circuit current density is more effective in the device using the DCB solvent than in the device using the CB solvent. In addition, for wavelengths ranging from 400 to 700 nm, the IPCE value for the device with DCB is higher than that of the device with CB, as shown in Figure 2b. The maximum IPCE of the devices with CB and DCB is ~66% and ~72% at ~510 nm, respectively. Using the AM1.5G reference spectrum, these values are converted to 9.07 and 9.97 mA/cm^2^, which are similar to the J_SC_ values of the device with CB and DCB, respectively. These results indicate that, for the inverted PSCs, the device with DCB is more suitable than the device using CB. 

**Figure 3:**
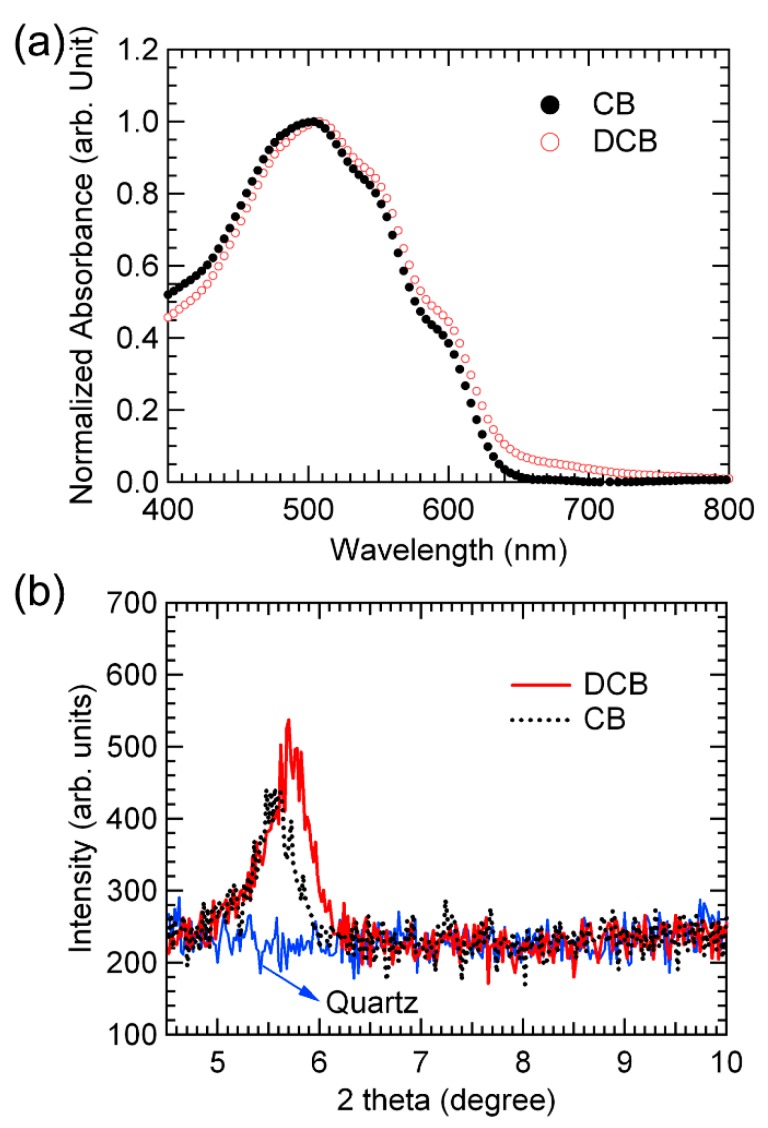
(**a**) UV-visible absorption spectra and (**b**) XRD patterns of P3HT:PCBM films using CB and DCB solvents on a quartz substrate.

The effect of CB and DCB solvents on the device performance was investigated by measuring the nanoscale morphology of each film using AFM, optical absorption spectra, XRD patterns, and electrical impedance spectroscopy. To compare the crystalline structure of P3HT:PCBM films prepared with different solvents, we analyzed the UV-visible absorption spectra and XRD profiles, as shown in Figure 3. The amount of generated charge in an organic solar cell is proportional to the absorption of light, and therefore the absorption properties affect the improvement of IPCE. This is because the IPCE refers to the efficiency with which photons absorbed by each wavelength are converted into photocurrent. As Figure 3a shows, absorption intensity above 500 nm associated with the use of DCB is stronger than that of CB, in which this result led to the improvement of the IPCE of the solar cell using DCB in Figure 2b and indicating that P3HT-conjugated chains form a more ordered phase when DCB is used. This is consistent with the stronger peak intensity in the XRD pattern obtained for the P3HT:PCBM film using DCB compared with that of the intensity obtained for the film using CB (see Figure 3b). The diffraction peaks of 2θ = 5.4° are observed regardless of the P3HT:PCBM solvent. This indicates that the P3HT backbone in the crystalline domain is mainly parallel to the substrate, whereas the side chains are perpendicular to the substrate.^11^ Thus, the stronger peak intensity obtained for the P3HT:PCBM film using DCB indicates that the degree of P3HT crystallization can be improved by using DCB rather than CB. This improved crystallinity of P3HT contributes to improvements in the J_SC_ and FF, as shown in Figure 2a, although the thickness of the DCB-based device is lower than that of the CB-based device.

**Figure 4:**
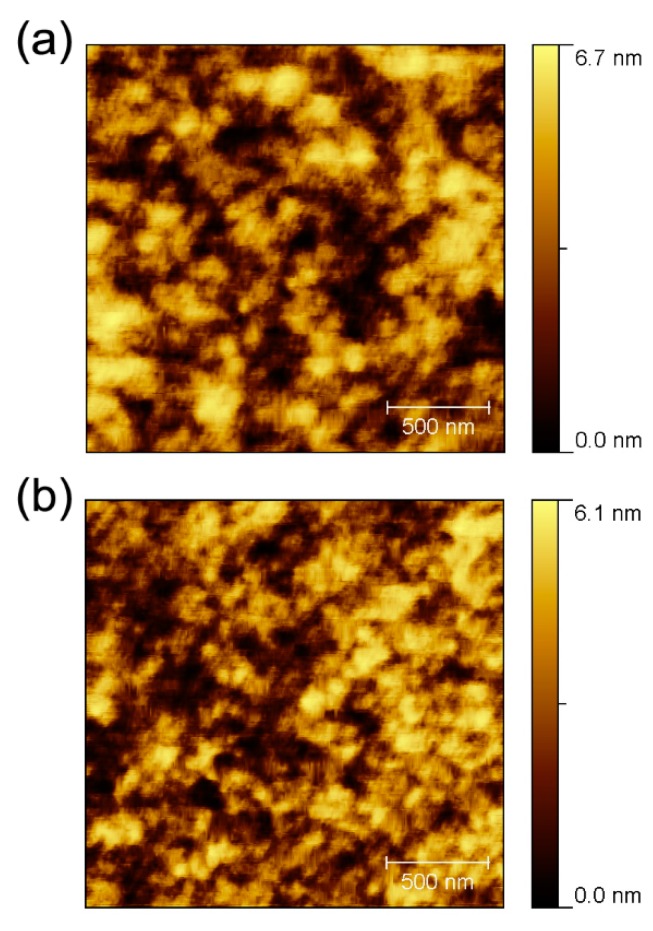
AFM morphology images of P3HT:PCBM thin films using (**a**) CB and (**b**) DCB solvents.

Figure 4 shows the surface morphology, as measured through a non-contact AFM method, of the P3HT:PCBM films using CB and DCB. The images indicate that the films are homogeneous, crack-free, and continuous with very well-connected grains. The root-mean square (RMS) roughness values of the P3HT:PCBM films using CB and DCB were 0.91 and 0.75 nm, respectively. The surface of the film using DCB is smoother than that of the film using CB. These results imply that the increased smoothness of the surface yields increased (i) interfacial contact between the P3HT:PCBM layer and the MoO_3_/Al layer, and (ii) separation of photo-generated electron and hole pairs at the P3HT:PCBM interface. However, the higher surface roughness may indicate non-uniform coverage, which may in turn affect the interface resistance. The improved performance of the device is attributed to the improvement in the morphology of the electron and hole separation in the photo active layer [27].

**Figure 5:**
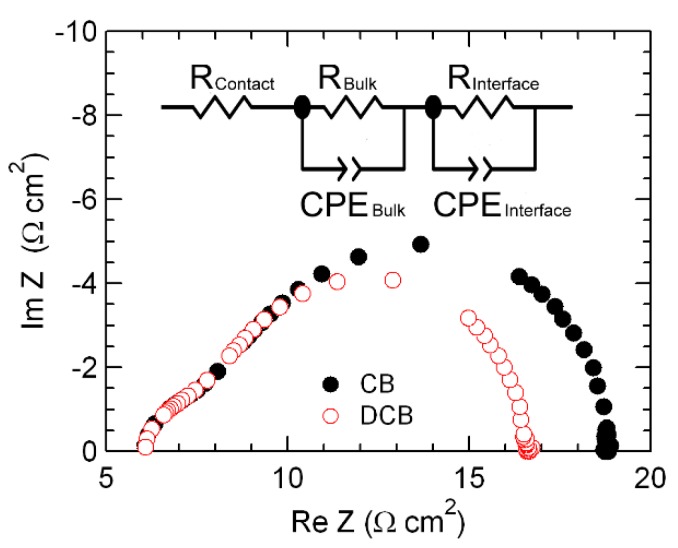
Cole–Cole plots of the devices using CB and DCB solvents under 100 mW/cm^2^ illumination. The plots are fitted by an equivalent circuit (a CPE model is shown in the inset).

Figure 5 shows the Cole–Cole plots of the devices using different solvents under 100 mW/cm^2^ illumination. They are divided by two semi-circles at a frequency of ~50 kHz and fitted by an equivalent circuit consisting of resistors with resistance (R) and constant phase elements (CPE) with capacitance for the contact, bulk, and interface characteristics (see inset in Figure 5). Organic materials have a non-ideal and frequency-dependent capacitance, due to the material inhomogeneity or grain boundaries. Therefore, compared with the RC model, the analysis using the CPE model is more suitable for the consideration of inverted PSCs [28,29].

(1) $$Z=Z^{\prime }+Z^{\prime \prime }=R_{C}+\frac{R_{B}+j\omega {R_{B}}^{2}C_{B}}{1+\omega^{2}{R_{B}}^{2}{C_{B}}^{2}}+\frac{R_{J}+j\omega {R_{I}}^{2}C_{I}}{1+\omega^{2}{R_{I}}^{2}{C_{I}}^{2}}$$

(2) $$Z^{\prime }=R_{C}+\frac{R_{B}}{1+\omega^{2}{R_{B}}^{2}{C_{B}}^{2}}+\frac{R_{I}}{1+\omega^{2}{R_{I}}^{2}{C_{I}}^{2}}$$

(3) $$Z^{\prime \prime }=\frac{\omega {R_{B}}^{2}C_{B}}{1+\omega^{2}{R_{B}}^{2}{C_{B}}^{2}}+\frac{\omega {R_{I}}^{2}C_{I}}{1+\omega^{2}{R_{I}}^{2}{C_{I}}^{2}}$$

where *R_C_* is contact resistance, *R_B_* is bulk resistance, *R_I_* is interface resistance, *C_B_* is bulk capacitance, and *C_I_* is interface capacitance, which are related to the series resistance of ITO, the P3HT:PCBM BHJ layer, and interfaces between the P3HT:PCBM layer and the MoO_3_/Al layer, respectively. As a result, *R_C_*, *R_B_*, and *C_B_* values in the high-frequency range (i.e., 6.3 Ω·cm^2^, 1.3 Ω·cm^2^, and 3.5 μF) are nearly the same for both solvents. However, for the low-frequency range, using CB yields higher R_I_ and C_I_ values (11.3 Ω·cm^2^ and 1.9 μF) than those obtained using DCB (9.0 Ω·cm^2^ and 1.2 μF). These results indicate that, for the device using DCB, the charge extraction property characterizing P3HT:PCBM and the charge carrier buffer layers is superior to that of the device using CB. This superior performance is attributed to the better P3HT crystallization and interface characteristics of the former compared to those of the latter. In other words, the extraction properties of the carriers generated from P3HT: PCBM would be better in the device using DCB than that of CB-based devices, which is why the interfacial resistance of DCB devices was measured to be lower. In addition, since the FF of the organic solar cell is highly related to the extraction properties [30,31], the FF of DCB-based device is higher than that of the CB-based device due to the difference in interface resistances.

## 4. Conclusions

We have investigated the effect of solvents for BHJ blend films of P3HT:PCBM. The volatility difference between CB and DCB solvents resulted in different P3HT crystallization and charge carrier extraction characteristics, leading to different device characteristics. The better P3HT crystallization of the P3HT:PCBM film using DCB (compared with that of the film using CB) increased the light absorption at longer wavelengths, owing to highly ordered conjugation chains. Moreover, the better charge carrier extraction of the device using the DCB solvent was evidenced by the smoother P3HT:PCBM surface morphology and lower interface impedance values, compared with those of the device using the CB solvent.
